# Characterization of flag leaf morphology identifies a major genomic region controlling flag leaf angle in the US winter wheat (*Triticum aestivum* L.)

**DOI:** 10.1007/s00122-024-04701-1

**Published:** 2024-08-14

**Authors:** Pradeep Kumar, Harsimardeep S. Gill, Mandeep Singh, Karanjot Kaur, Dante Koupal, Shyamal Talukder, Amy Bernardo, Paul St. Amand, Guihua Bai, Sunish K. Sehgal

**Affiliations:** 1 Department of Agronomy, Horticulture and Plant Science, South Dakota State University, Brookings, SD USA; 2 Department of Soil and Crop Sciences, Texas A&M University, Texas A&M AgriLife Research Center, Beaumont, TX USA; 3 USDA-ARS, Hard Winter Wheat Genetics Research Unit, Manhattan, KS USA

## Abstract

**Key message:**

Multi-environmental characterization of flag leaf morphology traits in the US winter wheat revealed nine stable genomic regions for different flag leaf-related traits including a major region governing flag leaf angle.

**Abstract:**

Flag leaf in wheat is the primary contributor to accumulating photosynthetic assimilates. Flag leaf morphology (FLM) traits determine the overall canopy structure and capacity to intercept the light, thus influencing photosynthetic efficiency. Hence, understanding the genetic control of these traits could be useful for breeding desirable ideotypes in wheat. We used a panel of 272 accessions from the hard winter wheat (HWW) region of the USA to investigate the genetic architecture of five FLM traits including flag leaf length (FLL), width (FLW), angle (FLANG), length–width ratio, and area using multilocation field experiments. Multi-environment GWAS using 14,537 single-nucleotide polymorphisms identified 36 marker-trait associations for different traits, with nine being stable across environments. A novel and major stable region for FLANG (*qFLANG.1A*) was identified on chromosome 1A accounting for 9–13% variation. Analysis of spatial distribution for *qFLANG.1A* in a set of 2354 breeding lines from the HWW region showed a higher frequency of allele associated with narrow leaf angle. A KASP assay was developed for allelic discrimination of *qFLANG.1A* and was used for its independent validation in a diverse set of spring wheat accessions. Furthermore, candidate gene analysis for two regions associated with FLANG identified seven putative genes of interest for each of the two regions. The present study enhances our understanding of the genetic control of FLM in wheat, particularly FLANG, and these results will be useful for dissecting the genes underlying canopy architecture in wheat facilitating the development of climate-resilient wheat varieties.

**Supplementary Information:**

The online version contains supplementary material available at 10.1007/s00122-024-04701-1.

## Introduction

Bread wheat (*Triticum aestivum*) is the most extensively grown staple cereal crop for ensuring the food security of the growing human population worldwide. It provides approximately 19% of the total dietary calories and 21% of proteins globally along with a wealth of additional nutrients that promote human health in everyday diets (Tadesse et al. 2019). Given that the global human population is projected to reach 10 billion by 2050 and that farmland will decline steadily, there will inevitably be a need to increase food production. However, current trends indicate that progress toward higher-yielding wheat cultivars may fall short of meeting the impending demand (Tilman et al. 2011; Ray et al. 2013). Therefore, enhancing the genetic potential of wheat productivity remains the primary objective for all wheat breeding programs.

Many studies have shown that the photosynthetic capacity of crop species will play a major role in improving crop yield (Zhu et al. 2008; Ort et al. 2015). Canopy architecture and photosynthesis efficiency together play a pivotal role in determining photosynthetic capacity; therefore, it is imperative to discover genes that control canopy architecture and sustained flow of nutrients that lead to optimal spike development and yield (Mantilla-Perez and Salas Fernandez 2017; Ma et al. 2020). The flag leaf plays an important role in wheat as the primary site for accumulating photo-assimilates contributing 40–60% of photosynthetic performance and over 40% of assimilates during the grain-filling stage (Sharma et al. 2003; Ba et al. 2020; Du et al. 2022). Various characteristics of flag leaf morphology including the size and area of the flag leaf have been found to be positively associated with various yield-related traits and ultimately contribute to wheat productivity (Fan et al. 2015; Liu et al. 2018a). Additionally, high yields have been associated with the size and area of the flag leaf in rice and have become a breeding target to achieve an ideal phenotype (Li et al. 1998; Zhang et al. 2014).

Flag leaf morphology (FLM) is characterized by the flag leaf length (FLL), width (FLW), angle (FLANG), area (FLA), and ratio (FLR), which together determine the canopy architecture. Understanding the genetic mechanisms governing these quantitative traits influenced by environmental factors will enable the development of genotypes with stable and enhanced photosynthetic efficiency (Kobayashi et al. 2003). Photosynthesis is affected by many factors that determine the distribution of light over the leaves in the canopy. Among the flag leaf traits, leaf posture, or more specifically leaf inclination (angle), plays an important role in terms of light interception over the canopy. The flag leaf angle (FLANG), defined as the angle between the leaf blade midrib and the stem, directly influences the amount of light that plants receive, which ultimately affects yield (Mantilla-Perez and Salas Fernandez 2017). Modeling approaches have been employed for decades to predict the optimal plant characteristics that would optimize yield with leaf inclination being a key factor (Mantilla-Perez and Salas Fernandez 2017). Many researchers have proposed that upright leaf angles in the upper canopy, less erect leaves in the middle canopy, and more horizontally oriented leaves in the lower canopy provide an ideal plant architecture (Long et al. 2006; Zhu et al. 2010; Ku et al. 2011; Ort et al. 2015). The impact of leaf angle on photosynthetic efficiency has been extensively studied in various cereal crops including maize (Hammer et al. 2009; Fischer and Edmeades 2010; Edwards 2011; Tian et al. 2011), rice (Sinclair and Sheehy 1999; Sakamoto et al. 2006; Kumagai et al. 2009), barley, sorghum, and oats (Tanner et al. 1966; Shearman et al. 2005; Fischer and Edmeades 2010; Truong et al. 2015). The findings of these studies suggested that cultivars with erect leaves exhibit better light interception, higher photosynthesis, greater crop growth, less photoinhibition, and higher yield in comparison with those with horizontally positioned leaves. The plant model for 'super' high-yielding hybrid rice, also known as the 'second generation of new plant types (NPTs),’ incorporates a reduced leaf angle as one of its suggested morphological characteristics (Li et al. 2002; Peng et al. 2008). In wheat, Donald (1968) also considered upright leaves as the desired 'ideotype.' Over the past five decades, studies in wheat have consistently suggested that genotypes with small and erect leaves exhibit higher exposure of leaf area to sunlight resulting in higher Leaf Area Index (LAI) and a higher rate of dry matter production, which ultimately translates to superior yields (Donald 1968; Choudhury 2000; Parry et al. 2011). In rice, narrow flag leaf angles have been reported to result in 13% higher photosynthetic rates, a reduction in photoinhibition, and 15% higher yields (Chen et al. 2020; Mantilla-Perez and Salas Fernandez 2017). Thus, understanding the underlying genetics of leaf angle is crucial for the development of photosynthetically efficient wheat cultivars.

Several studies have been conducted to characterize the FLM traits in wheat. Most of these studies used linkage mapping to identify QTLs governing FLM traits (Xue et al. 2013; Fan et al. 2015; Yang et al. 2016; Hussain et al. 2017; Zhao et al. 2018; Liu et al. 2018a, b; Khanna-Chopra et al. 2020; Ma et al. 2020; Yan et al. 2020a, b, c; Wang et al. 2022). However, the majority of these studies have focused on the FLL and FLW, and few studies have focused on the FLANG (Yang et al. 2016; Sun et al. 2017; Liu et al. 2018a; Chen et al. 2021). Liu et al. (2019) used map-based cloning to identify the first gene *TaSPL8* that regulates leaf angle in wheat. *TaSPL8* encodes a SQUAMOSA PROMOTER BINDING-LIKE (SPL) protein that regulates leaf angle in wheat through auxin signaling and brassinosteroid biosynthetic pathway. Recently, Zhang et al. (2024) fine-mapped and validated a major quantitative trait locus on chromosome 4B for flag leaf angle in wheat and identified candidate genes underlying this QTL using expression analysis. It is also noteworthy that only a handful of studies have used genome-wide association studies (GWAS) to characterize FLM traits in wheat, especially FLANG. Most of these studies have focused on the characterization of yield-related traits using FLM traits as secondary traits ( Sun et al. 2017; Liu et al. 2017; Chen et al. 2021; Li et al. 2021; Muhammad et al. 2021a, b; Wang et al. 2022). Furthermore, the majority of reported studies are from China, whereas such studies have not been reported in the US winter wheat. In the above context, there is an opportunity to explore the phenotypic and genetic diversity associated with FLM traits in US winter wheat that will provide insights into the genetic mechanism governing FLM traits and enable its utilization in wheat breeding programs.

The majority of GWAS reports in various crop species commonly utilize assembled diversity panels or landraces (Sidhu et al. 2020; AlTameemi et al. 2021; Gudi et al. 2024), rather than using the breeding materials (Ward et al. 2019). The existing genotypic variation in various germplasm and diversity panels provides an opportunity to exploit photosynthetic efficiency through characterization and subsequent integration into breeding programs (Fischer and Edmeades 2010; Lawson et al. 2012; Singh et al. 2022). However, the utilization of elite breeding lines within these panels not only assists in uncovering novel genomic regions associated with the desired trait(s) but also enables the successful integration of beneficial alleles into the cultivar development process without any linkage drag (Gill et al. 2022).

To the best of our knowledge, this is the first study on the characterization of FLM traits in US wheat germplasm in multilocation field experiments. We assembled a population comprising diverse lines from the South Dakota State University (SDSU) breeding program and a set of released cultivars/breeding lines from different breeding programs in the US Great Plains region. The assembled panel was genotyped using genotyping-by-sequencing (GBS) and evaluated for FLM traits in field trials at multiple locations. The objectives of the current study were to (a) assess the phenotypic and genetic variation for the FLM traits in the US hard winter wheat, (b) dissect the genetic architecture of these traits using GWAS to identify genomic regions and putative candidate genes for the FLM using a wheat reference genome, and (c) develop and validate Kompetitive Allele Specific Polymerase Chain Reaction (KASP) assays for important genomic regions to facilitate marker-assisted selection (MAS) and fine mapping.

## Materials and methods

### Plant materials and experimental setup

In this study, we assembled a new association mapping panel named the Winter Wheat Association Mapping (WWAM) panel, which includes 272 diverse winter wheat accessions (Supplementary Table S1). The WWAM panel primarily comprised breeding lines developed over the last 10 years at the South Dakota State University (SDSU) winter wheat breeding program, selected based on their diverse pedigrees. To enhance mapping resolution, the panel also included historical and recently released cultivars and elite lines from the Hard Winter Wheat (HWW) region that have been widely used in various breeding programs. Additionally, a small set of winter wheat accessions from the global collection of wheat lines, which were recently exome-captured (He et al. 2019), were included in the panel based on their previous evaluation in the South Dakota environment. The panel also included ‘Kharkof’ and ‘Scout-66’ which have been used as historical checks in regional HWW nurseries. The metadata for the WWAM panel is provided in Supplementary Table S1.

The WWAM panel was evaluated for FLM traits in three field environments including SDSU experimental stations at Aurora, Brookings, and Felt farm, South Dakota (referred to as E1, E2, and E3) in the 2022–2023 winter wheat growing season. Each experiment was planted using a randomized complete block design (RCBD) with two replications (complete blocks), resulting in six independent field replicates for the whole experiment. The experimental unit within each replicate was a four-row plot that was 1.25 m long with an inter-row spacing of 20 cm. All the trials were managed following regional standard cultural practices for proper growth and development of wheat.

### Phenotyping for FLM and agronomic traits

After flowering, disease-free flag leaves from the 10 main tillers in the two middle rows of each plot were selected for measuring the flag leaf length (FLL), flag leaf width (FLW), and flag leaf angle (FLANG). Two additional traits, namely flag leaf area (FLA) and the length-to-width ratio of the flag leaf (FLR), were estimated as described in previous studies (Ma et al. 2020; Chen et al. 2021). The FLL was determined by measuring the distance from the base to the tip of the leaf, while flag leaf width (FLW) represented the width at the broadest section of the leaf. The FLANG was measured as the angle between the stem and midrib of the blade. FLM measurements were taken after flowering (Feekes 10.5.1), with the identification and tagging of ten plants 4–5 days post-flowering using spray paint to ensure consistency in the crop stage in each plot. The flag leaf area was measured as the multiplication of FLL and FLW by a factor of 0.75 (FLL × FLW × 0.75) (Yang et al. 2016; Liu et al. 2018b). Plant height (PH) was measured as the distance from the base of the stem to the top of the spike, excluding the awns after reaching physiological maturity. Days to heading (DH) were recorded as the Julian days when 50% of the heads were emerged in each plot.

### Statistical analysis of phenotype data

The statistical analyses were performed using various packages in R environment (Core R Team 2019). Initially, we analyzed the three experiments independently to obtain the best linear unbiased estimates (BLUE) for various traits using the following model:

$${y}_{ij} = \mu + {R}_{i}+ {G}_{j} + {e}_{ij}$$

where ${y}_{ij}$ is the trait of interest, $\mu$ represents the overall mean, ${R}_{i}$ denotes the random effect of the *i*^*th*^ replicate in each location, ${G}_{j}$ refers to the fixed effect of the *j*^*th*^ genotype, and ${e}_{ij}$ refers to the residual error effect of the *i*^*th*^ replication and *j*^*th*^ genotype.

Secondly, BLUEs were calculated from all three environments using the following equation:

$${y}_{ijk} = \mu + {E}_{i}+ {R}_{j(i)}+ {G}_{k}+{ GE}_{ik} + {e}_{ijk}$$

where ${y}_{ijk}$ is the trait of interest, $\mu$ is overall mean, ${E}_{i}$ refers to the random effect of the *i*th environment, ${R}_{j(i)}$ refers to the random effect of the *j*th replicate nested in the *i*th environment, ${G}_{k}$ refers to the fixed effect of the *k*th genotype, ${GE}_{ik}$ denotes the genotype by environment (*G *× *E*) interaction, and ${e}_{ijk}$ refers to the residual error. The environment corresponds to the individual locations, and replicates correspond to the complete blocks within each location.

The broad-sense heritability (*H*^2^) was estimated by fitting the genotypic effect from the above equation as random using the following formula:

$${H}^{2}= \frac{{\sigma }_{g}^{2}}{{\sigma }_{g}^{2}+\frac{{\sigma }_{ge}^{2}}{n\text{Loc}}+\frac{{\sigma }_{e}^{2}}{n\text{Loc} \text{x} n\text{Rep}}}$$

where ${\sigma }_{g}^{2}$ and ${\sigma }_{e}^{2}$, are the genotype and error variance components, respectively; ${\sigma }_{ge}^{2}$ is the variance component for G × E, *n*Rep refers to the number of replicates, and *n*Loc refers to the number of locations. The above analysis was performed with the META-R tool (Alvarado et al. 2019), which uses ‘lme4’ package (Bates et al. 2015). The correlations among traits were obtained using the BLUEs from across environment analysis and visualized using ‘psych’ library in R (William 2023). All the model comparisons were visualized using ‘ggplot2’ in a R package (Wickham 2016).

### Genotyping, population structure, and linkage disequilibrium

The WWAM panel was genotyped using GBS as previously described (Gill et al. 2022) in different sequencing runs as a limited number of lines are sequenced each year for the breeding program. DNA was isolated from fresh leaf tissue collected at the three-leaf stage using the hexadecyltrimethylammonium bromide (CTAB) method (Doyle and Doyle 1987), and GBS libraries were prepared using the *PstI* and *MspI* restriction enzymes as described by Poland et al. (2012). The libraries were sequenced using Ion Proton sequencer (Thermo Fisher Scientific, Waltham, MA, USA) or NextSeq2000 sequencer (Illumina, San Diego) at the USDA Central Small Grain Genotyping Lab, Manhattan, KS. The raw GBS reads were used to call single-nucleotide polymorphisms (SNPs) for the complete set of 272 accessions using GBS discovery pipeline v2.0 in TASSEL v5.0 (Trait Analysis by Association, Evolution and Linkage) (Bradbury et al. 2007), and Chinese Spring (CS) wheat genome RefSeq v2.1 (Zhu et al. 2020) was used as the reference. The SNPs with > 30% missing data points, > 10% heterozygotes, < 5% minor allele frequency (MAF), or unmapped to any chromosome were removed for quality control, followed by imputation for missing data using BEAGLE v4.1 (Browning and Browning 2007). In addition, two accessions were removed due to the high frequency of missing data. After filtration, 14,537 high-quality SNPs for 270 accessions were retained and used for further analysis.

The WWAM panel was examined for population stratification using principal component analysis (PCA) and STRUCTURE analysis. PCA was performed based on the genotypic data using ‘SNPRelate’ package (Zheng et al. 2012) and visualized using ‘ggplot2’ in R (Wickham 2016). A Bayesian model-based clustering program, STRUCTURE v2.3.4, was also used assuming an Admixture model (Pritchard et al. 2000). We performed ten independent runs with a burn-in period of 20,000 iterations followed by 20,000 Monte Carlo iterations assuming ten subpopulations (*K* = 1–10). The analysis was performed in parallel using StrAuto v1.0 on the SDSU high-performance computing (HPC) cluster (Chhatre and Emerson 2017; Tange 2018). The most likely number of subpopulations within the WWAM panel was inferred using an ad hoc statistic (DeltaK) that used the rate of change in the log probability between runs using successive K-values using STRUCTURE HARVESTER (Evanno et al. 2005; Earl and vonHoldt 2012). Further, a neighbor-joining-based phylogenetic tree was constructed in TASSEL using the filtered set of SNP markers. Linkage disequilibrium (LD) analysis was performed using the complete set of SNPs with all pairwise marker comparisons and a sliding window size of 50 SNPs in TASSEL v5.0 (Bradbury et al. 2007). The LD decay for the whole genome as well as individual sub-genomes was obtained by fitting a nonlinear model using the modified Hill and Weir method (Hill and Weir 1988) as described in Gill et al. (2022) and visualized in the R.

### GWAS and candidate gene analysis

We performed GWAS using 14,537 SNPs and trait BLUEs obtained by analyzing individual experiments (E1, E2, and E3) as well as the BLUEs from combined analysis (CEnv). A multi-locus model, Fixed and random model Circulating Probability Unification (FarmCPU), was used to perform GWAS for all the traits (Liu et al. 2016). The FarmCPU model used four principal components to account for the population structure and was implemented in Genomic Association and Prediction Integrated Tool (GAPIT) version 3.0 in the R (Wang and Zhang 2021). The fit of the model was observed by visualizing the quantile–quantile (QQ) plots. A stringent criterion was set to report the marker-trait associations (MTAs) that were stable and observed in different environments. According to this criterion, only those stable MTAs were reported that were identified in at least three GWAS analyses (E1, E2, E3, or CEnv) at an exploratory threshold of − log10(*P*) = 3.50, as well as surpassed the Bonferroni-corrected (*P* < 0.05) threshold of − log_10_(*P*) = 5.40 in one of those three environments*.* The proportion of the phenotypic variance explained by a major genomic region on chromosome 1A associated with FLANG (*qFLANG.1A*) was estimated by fitting a mixed-linear model (MLM) in TASSEL v5.0 (Bradbury et al. 2007). We performed a pairwise comparison of two alleles of *qFLANG.1A* identified on chromosome 1A, for differences in FLANG, days to heading, and plant height. For this purpose, mean trait values (BLUEs from combined analysis) for two groups of alleles were compared using a *t-test* and visualized using boxplots in R.

We performed candidate gene analyses for the selected regions based on the local LD, rather than relying on a random upstream/downstream search around the significant SNP. For candidate gene analyses of identified genomic regions, we used rTASSEL (Monier et al. 2021) to visualize LD blocks in the region harboring associated SNP. Further, we used CS RefSeq v2.1 (Zhu et al. 2021) to extract all high confidence (HC) genes within the LD block harboring respective SNP. The coding sequences for HC genes were retrieved from the CS RefSeq v2.1 database and were used to manually annotate the HC genes using ‘Blast2GO’ (Conesa et al. 2005). The functional annotation, comparison with reported FLM-related genes, and publicly available expression databases were used to select the putative candidate genes of interest.

### Development of KASP and independent validation of *qFLANG.1A* on chromosome 1A

For the utilization of stable and novel genomic region for FLANG (*qFLANG.1A*) identified on chromosome 1A, we designed primers for the KASP assay. The 200 bp sequences from upstream and downstream of significant SNP associated with FLANG were identified in IWGSC CS RefSeq v2.1 (Zhu et al. 2021) to design KASP primers using PolyMarker (Ramirez-Gonzalez et al. 2015). The tail sequences were added to 5’-ends of the two forward primers and the tail sequences matched with FAM (6-carboxy-fluorescein) and HEX (Hexachlorofluorescein) fluorescence-labelled sequences in KASP mix (Supplementary Table S2). The total reaction volume for each KASP assay was 10.14 µL comprising 5 µL of genomic DNA (~ 50 ng), 5 µL of 2X KASP-TF master mix, and 0.14 µL of KASP assay mix. The details of KASP genotyping thermal cycle protocol are provided in Supplementary Table S3. The PCR and fluorescence signal detection were performed in the CFX96 Touch Real-Time PCR Detection System (Bio-Rad, USA). The KASP primers were first tested using a set of 158 lines selected from the WWAM panel representing two alleles.

Further, the developed KASP marker was used for independent validation of the qFLANG.1A in spring wheat to broaden the scope of findings from the current study. We used an independent natural population of 200 diverse spring wheat accessions randomly selected from the National Small Grains Collection (Supplementary Table S4). Importantly, the 200 accessions were part of a larger core collection that represents considerable diversity for spring wheat germplasm. The selected 200 accessions were grown in the greenhouse conditions using DEEPOT D40 CELLS (Stuewe & Sons, Tangent, OR, USA) filled with Sunshine R 360 potting soil (Sun Gro Horticulture, Agawam, MA, USA). The cells were arranged in trays (Stuewe & Sons, Tangent, OR, USA) following a randomized complete block design with three replications. The independent set was phenotyped for FLANG as described in the previous section. Further, the 200 accessions were subjected to KASP assay to determine the *qFLANG.1A* allele, and the average phenotypic data for FLANG were compared between two allelic groups of *qFLANG.1A* using a *t-test* and visualized using boxplots in R.

## Results

### Phenotypic variation, correlations, and heritability

**Fig. 1:**
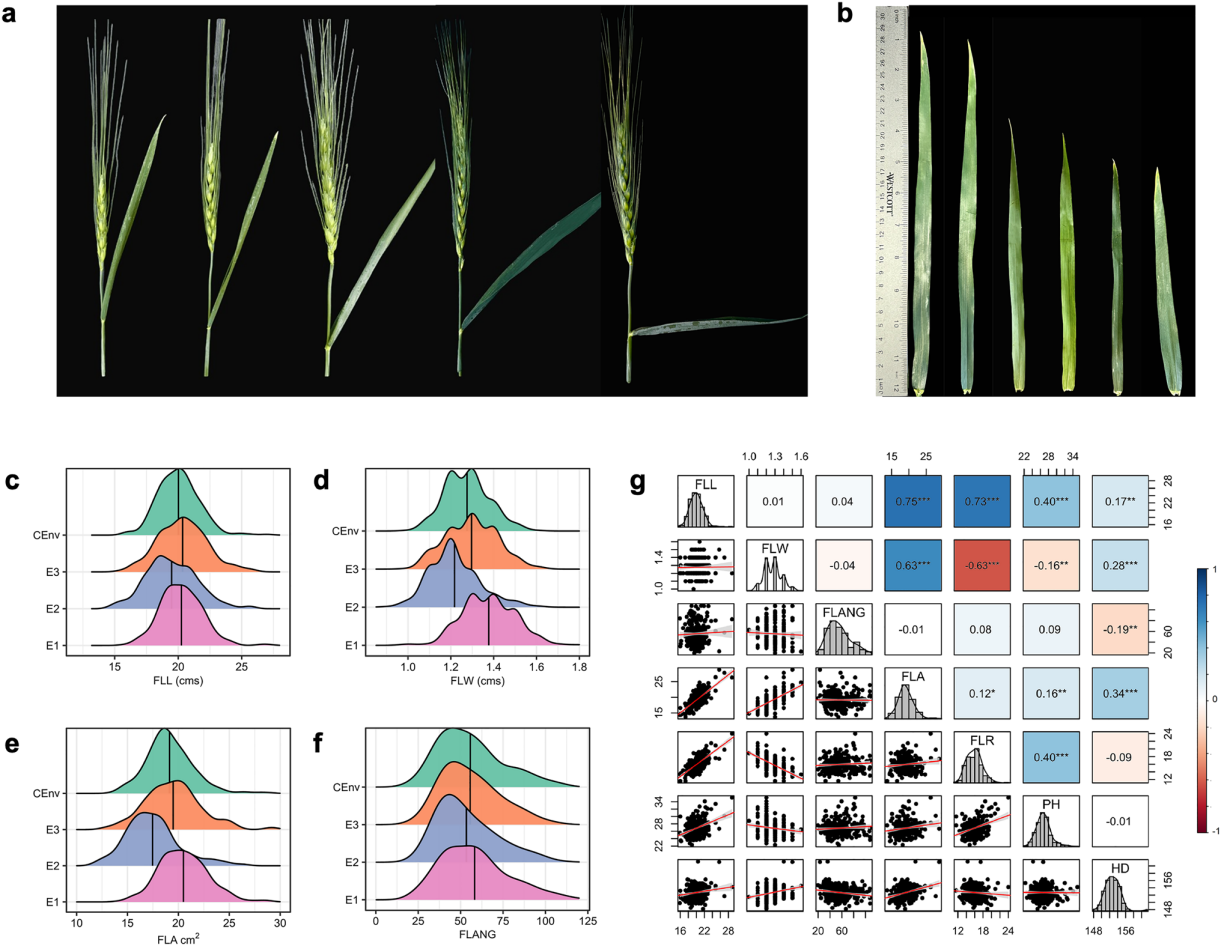
Phenotypic variability and correlation among various flag leaf morphology (FLM) traits in 272 genotypes of WWAM panel evaluated in three different environments (E1, E2, and E3) and combined over environments (CEnv). **a** Representative images of diverse phenotypes of FLANG, flag leaf angle; **b** FLL, flag leaf length. Density plots showing the phenotypic distribution of (**c**) FLL, flag leaf length; **d** FLW, flag leaf width; **e** FLA, flag leaf area; **f** FLANG, flag leaf angle. **g** Correlation coefficients among investigated traits were calculated using the best linear unbiased estimates (BLUEs) from a combined analysis of three environments. The pair plot shows bivariate scatter plots below the diagonal, histograms on the diagonal, and Pearson correlation between given traits above the diagonal. Statistically significant correlations are denoted by an asterisk (*) where * P ≤ 0.05, ** P ≤ 0.01, and *** P ≤ 0.001

A significant variation was observed for FLM traits in all tested environments (Table 1). The distribution for FLM traits was almost consistent in all three environments, with slightly lower FLW and FLA measurements in E2 compared to other environments (Figs. 1c-1f). A high broad-sense heritability (H^2^) was observed for FLM traits, ranging from 0.80 to 0.94 (Table 1) with the highest H^2^ (0.94) for FLANG. There was no significant correlation among three major FLM traits, including FLL, FLW, and FLANG (Fig. 1g). As expected, significant correlations were observed for FLA and FLR with FLL and FLW owing to the mathematical relationship between these traits (Fig. 1g). Plant height showed a significant positive correlation with FLL (*r* = 0.40, *P* ≤ 0.001), a nonsignificant correlation with FLANG (0.09), but a significant negative correlation with FLW (*r* = − 0.16, *P* ≤ 0.01). Days to heading showed lower but significant positive correlations with both FLL (*r* = 0.17, *P* ≤ 0.01) and FLW (*r* = 0.28, *P* ≤ 0.001), and a significant negative correlation with FLANG (*r* = − 0.19, *P* ≤ 0.01).

**Table 1:** Descriptive statistics for flag leaf traits and broad-sense heritability estimates were obtained using a combined analysis of three environments (CEnv)

Trait^s^	Mean	Min	Max	SD	CV	Heritability
FLL (cm)	20.0	15.9	28.9	1.7	6.14	0.82
FLW (cm)	1.3	1.0	1.6	0.1	7.23	0.81
FLANG (degree)	55.7	19.8	107.7	19.0	14.88	0.94
FLA (cm^2^)	19.1	13.7	28.8	2.3	10.73	0.80
FLR (ratio)	15.9	11.6	24.0	1.8	9.17	0.83
PH (inches)	26.9	22.3	35.1	1.9	7.84	0.79
HD (Julian days)	152.7	148.2	161.2	1.7	1.10	0.80

^s^FLL, flag leaf length; FLW, flag leaf width; FLANG, flag leaf angle; FLA, flag leaf area; FLR, flag leaf ratio; PH, plant height; HD, days to heading; CV, coefficient of variation; SD, standard deviation; Min, minimum; Max, maximum

### Population structure and linkage disequilibrium

**Fig. 2:**
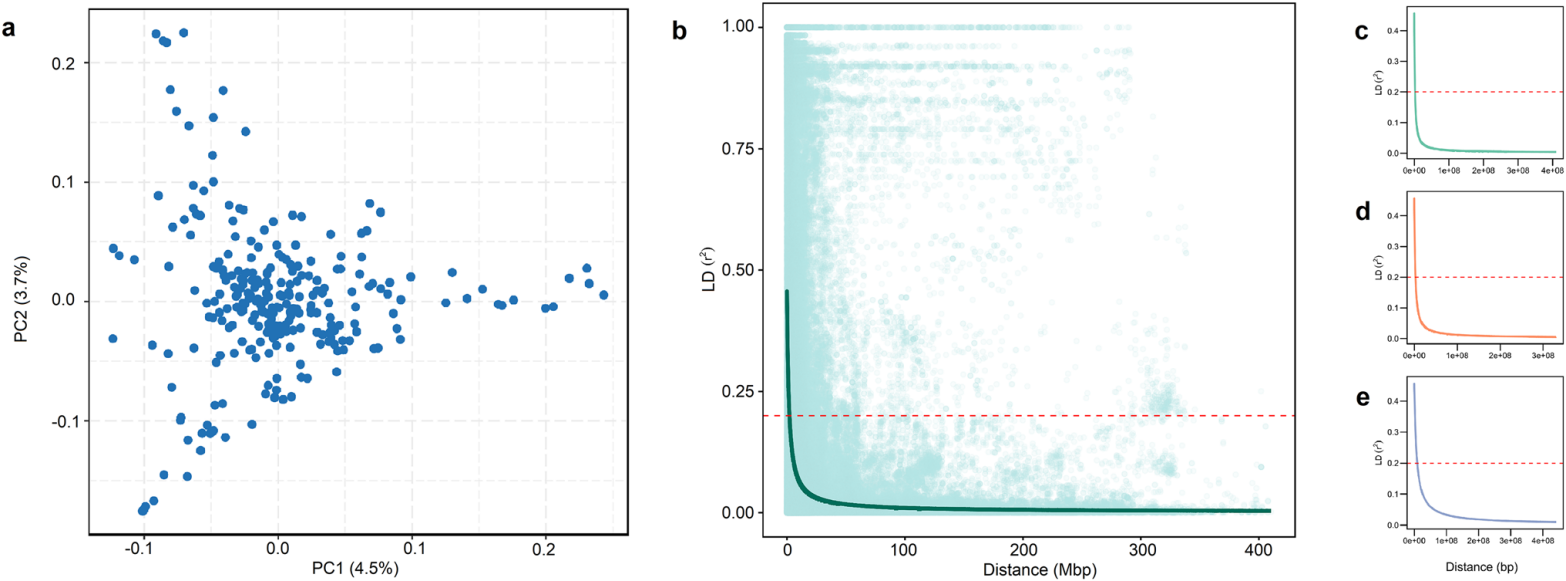
**a** The biplot for the first two components derived from the principal component analysis (PCA) of 270 genotypes based on 14,537 SNPs. Intra-chromosomal linkage disequilibrium (LD) in the WWAM panel for the whole genome (**b**), for A sub-genome (**c**), for B sub-genome (**d**), and for D sub-genome (**e**)

A set of 14,537 high-quality SNPs was used for analyzing the population structure and LD. Among the three sub-genomes, the highest number of SNPs were mapped to the B sub-genome (*n* = 6409), followed by A (*n* = 5641) and D (*n* = 2487) (Supplementary Table S5). Among chromosomes, chromosome 7A had the most SNPs (*n* = 1186), while chromosome 4D had the least SNPs (*n* = 117) (Supplementary Table S5). The STRUCTURE analysis showed a distinct peak at K = 4, suggesting the presence of four subgroups within the WWAM panel (Supplementary Figure S1). The principal component analysis also showed stratification in the WWAM panel, with the first two principal components explaining only 4.5% and 3.7% variance, respectively (Fig. 2a). To better explain the clustering within the WWAM panel, we used a neighbor-joining tree based on the genotypic data of the 270 lines (Supplementary Figure S2). Overall, the sub-groups within the WWAM panel were not very distinct. Two smaller sub-groups were identified with the first comprising of the germplasm derived from CIMMYT or Kansas material, and the second was dominated by material from Eastern Kansas and Nebraska. A third large sub-group comprised of majority SDSU releases and breeding lines, and it can be further divided into two groups with one having lines derived from Western Great Plains material, while the other one dominated by lines derived from Northern Plains material. The average LD decay distance was approximately 3 Mbp for the whole wheat genome (Fig. 2b) with a considerable variation in LD decay distance among the three sub-genomes (Figs. 2c-2e), with 1.7, 2.2, and 7 Mbp for sub-genomes A, B, and D, respectively.

### Association mapping for FLM traits

**Fig. 3:**
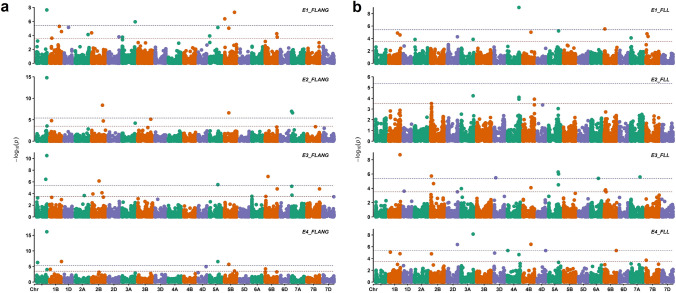
Manhattan plots presenting the results of genome-wide association studies for (**a**) flag leaf angle (FLANG) and (**b**) flag leaf length (FLL). The positions of SNPs on chromosomes are indicated on the x-axis relative to their -log_10_(*P*) values on the y-axis. The red dotted line refers to the exploratory threshold of -log_10_(*P*) = 3.5, and the blue dotted line refers to the Bonferroni-corrected threshold of -log_10_(*P*) = 5.40

The GWAS was conducted for five FLM traits (FLL, FLW, FLANG, FLA, and FLR) using BLUEs for each trait obtained from individual environments (namely E1, E2, and E3), as well as for the combined analysis (CEnv). In total, we identified 36 MTAs (Supplementary Table S6) for different FLM traits based on the Bonferroni-corrected threshold in individual GWAS analyses (Fig. 3). Nevertheless, we were interested in reporting only those MTAs that could be considered reliable based on stability in multiple environments. Hence, we focused on MTAs that were identified in at least three GWAS analyses (E1, E2, E3, or CEnv) at an exploratory threshold of − log_10_(*P*) = 3.50, as well surpassed the Bonferroni-corrected (*P* < *0.05*) threshold of − log_10_(*P*) = 5.40 in one of those three environments*.* Based on these criteria, a total of nine MTAs were identified representing nine QTLs for FLM traits (Table 2), with six for FLL and three for FLANG. Stable MTAs were not identified for FLW, FLA, and FLR (Table 2, Supplementary Table S7).

**Table 2:** Stable marker-trait associations (MTAs) identified in multiple environments (CEnv and at least two individual environments) for flag leaf length (FLL) and flag leaf angle (FLANG) identified by genome-wide association studies (GWAS) in WWAM panel

Trait^a^	MTA ID^b^	SNP^c^	Allele	Chromosome	Position^d^	MAF^e^	Log_10_(*P*)^f^	Env^g^
FLL	*qFLL.1B*	*S1B_653531690*	C/T	1B	653,531,690	0.27	4.81–8.96	E1, E3, CEnv
	*qFLL.2B*	*S2B_44931079*	C/T	2B	44,931,079	0.09	3.51–5.71	E2, E3, CEnv
	*qFLL.3A*	*S3A_688732686*	G/A	3A	688,732,686	0.08	3.85–8.15	E1, E2, CEnv
	*qFLL.4A*	*S4A_665013137*	T/A	4A	665,013,137	0.10	4.09–8.99	E1, E2, CEnv
	*qFLL.4B*	*S4B_459361891*	G/C	4B	459,361,891	0.31	4.08–6.41	E1, E3, CEnv
	*qFLL.5A*	*S5A_570625938*	G/C	5A	570,625,938	0.05	4.48–6.00	E1, E3, CEnv
FLANG	*qFLANG.1A*	*S1A_581955638*	C/G	1A	581,955,638	0.32	7.64–16.13	E1, E2, E3, CEnv
	*qFLANG.5A*	*S5A_473723888*	G/C	5A	473,723,888	0.08	5.12–6.56	E1, E3, CEnv
	*qFLANG.5B*	*S5B_247555050*	G/A	5B	247,555,050	0.09	5.02–6.60	E1, E2, CEnv

^a^FLL, flag leaf length; FLANG, flag leaf angle

^b^The column contains an ID used to represent each of the stable marker-trait association (MTA) or associated genomic region

^c^Single nucleotide polymorphism with the peak threshold value

^d^Physical position is based on IWGSC RefSeq v2.1 (IWGSC 2018; Zhu et al. 2021)

^e^Minimum allele frequency

^f^The range for threshold depicts the minimum to maximum -log10(P) values obtained by GWAS in different environments

^g^The environment(s) where the MTA was declared significant based on the described threshold

For FLL, six stable MTAs were identified on chromosomes 1B, 2B, 3A, 4A, 4B, and 5A, with all six MTAs being detected in three GWAS analyses (Table 2, Fig. 3b). The most significant association for FLL was detected on chromosome 1B (represented by SNP *S1B_653531690*) with − log_10_(*P*) values ranging from 4.81 to 8.96. Based on a comparison with previous reports, three of the six MTAs for FLL mapped on chromosomes 1B, 4A, and 4B represented novel genomic regions for this trait. Furthermore, three novel stable MTAs were detected for FLANG located on chromosomes 1A, 5A, and 5B (Table 2, Fig. 3a). Among the three MTAs for FLANG, a major genomic region (represented by SNP *S1A_581955638*) on the long arm of chromosome 1A (referred to as *qFLANG.1A*) was of particular interest owing to its consistency in all environments and strong signal. The *qFLANG.1A* (mapped at 581.9 Mbp on chromosome 1A) was detected in all four GWAS analyses with a strong signal, with − log_10_(*P*) ranging from 7.64 in E1 to 16.13 in CEnv. Moreover, the percentage of variance explained by *qFLANG.1A* (*S1A_581955638*) was estimated by fitting an MLM model and it ranged from 9 to 13% in different environments.

**Fig. 4:**
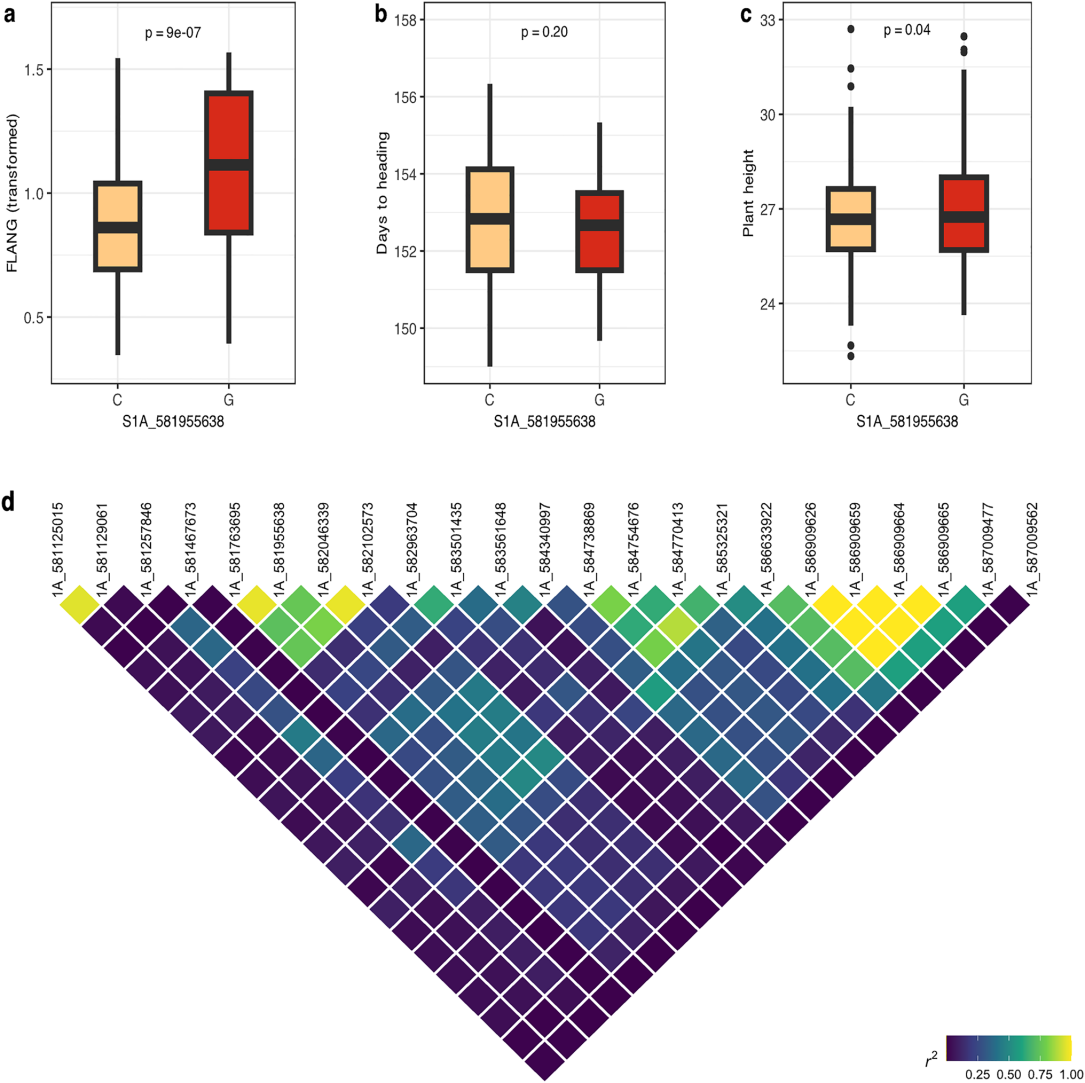
Boxplots showing the effect of two alleles of the MTA *qFLANG.1A* (*S1A_581955638*) on the trait means for (**a**) flag leaf angle (FLANG), **b** days to heading (HD), and **c** plant height (PH). **d** Heatmap presenting the local linkage disequilibrium in the region harboring *qFLANG.1A*

Further, we performed a pairwise comparison of two alleles of SNP *S1A_581955638,* which represents *qFLANG.1A* to evaluate the allelic effects on FLANG, PH, and HD. The 270 accessions in the WWAM panel were grouped based on the *S1A_581955638* allele (C/G), and the two groups were compared for mean trait values using a t-test. The two groups significantly differed (*P* < 0.001) in FLANG, but not in plant height or days to heading (Fig. 4a-4c).

### Candidate gene analysis

Candidate gene analysis was performed for only two genomic regions for FLANG (*qFLANG.1A* and *qFLANG.5A*), as LD blocks were notably larger (> 7 Mbp) for the other seven regions associated with FLL and FLANG. The two genomic regions were mapped on IWGSC CS RefSeq v2.1, and high-confidence genes were identified from the candidate region based on the available wheat expression data and previous literature. For *qFLANG.1A*, we visualized the LD around the associated SNP and did not observe a clear single LD block (Fig. 4d). Hence, we extracted the high-confidence (HC) genes from the ~ 581 to ~ 587 Mbp region on chromosome 1A. Out of a total of 79 HC genes from this region, seven putative candidate genes were identified encoding various types of proteins including a F-box family protein, kinase domains, and a glucan endo-1,3-beta-glucosidase 3-like protein (Table 3). Furthermore, the significant SNP (*S1A_581955638*) for *qFLANG.1A* was mapped within the gene *TraesCS1A03G1030200* encoding glucan endo-1,3-beta-glucosidase 3. Although we did not find any predicted protein in the candidate region that was previously reported to regulate FLM in the literature, it would be valuable to further investigate the gene underlying *qFLANG.1A.*

**Table 3:** List of putative candidate genes of interest along with their functional annotation identified in the regions harboring two genomic regions associated with FLANG on chromosomes 1A and 5A in wheat

MTAs	Chromosome	Gene ID^a^	Start Position	Functional annotation
*qFLANG.1A*	1A	*TraesCS1A03G1026000*	580,616,384	F-box family protein
	1A	*TraesCS1A03G1029600*	581,733,318	Glucan endo-1,3-beta-glucosidase 3
	1A	*TraesCS1A03G1029800*	581,762,132	Receptor-like protein kinase, putative, expressed
	1A	*TraesCS1A03G1029900*	581,769,626	Receptor-like protein kinase, putative, expressed
	1A	*TraesCS1A03G1030100*	581,943,225	Receptor-like protein kinase, putative, expressed
	1A	*TraesCS1A03G1030200*	581,953,050	Glucan endo-1,3-beta-glucosidase 3
	1A	*TraesCS1A03G1042900*	586,711,904	Protein SPA1-RELATED 3
*qFLANG.5A*	5A	*TraesCS5A03G0644800*	473,924,558	SHAGGY-like kinase
	5A	*TraesCS5A03G0653500*	476,708,174	zinc finger protein WIP2-like
	5A	*TraesCS5A03G0653900*	476,899,675	serine/threonine-protein kinase UCN-like
	5A	*TraesCS5A03G0656300*	477,320,230	transcription factor bHLH130-like
	5A	*TraesCS5A03G0657100*	477,704,015	FT-interacting protein 7
	5A	*TraesCS5A03G0657600*	477,918,048	ETHYLENE INSENSITIVE 3-like 3 protein
	5A	*TraesCS5A03G0658100*	478,065,316	squamosa promoter-binding-like protein

^a^GeneID and position of gene are based on IWGSC CS RefSeq v2.1

Further, 64 HC genes were retrieved in the *qFLANG.5A* region (473–479 Mb) on chromosome 5A, and seven were identified as putative candidates based on their annotations (Table 3). Among these seven genes, *TraesCS5A03G0658100 and TraesCS5A03G0644800* encoding SHAGGY-like kinase and a squamosa promoter-binding like (SPL) protein, respectively, are of particular interest because of their involvement in brassinosteroid (BR) pathway or relatedness to previously characterized genes for FLANG.

### Spatial distribution of *qFLANG.1A* alleles in HWW germplasm

**Fig. 5:**
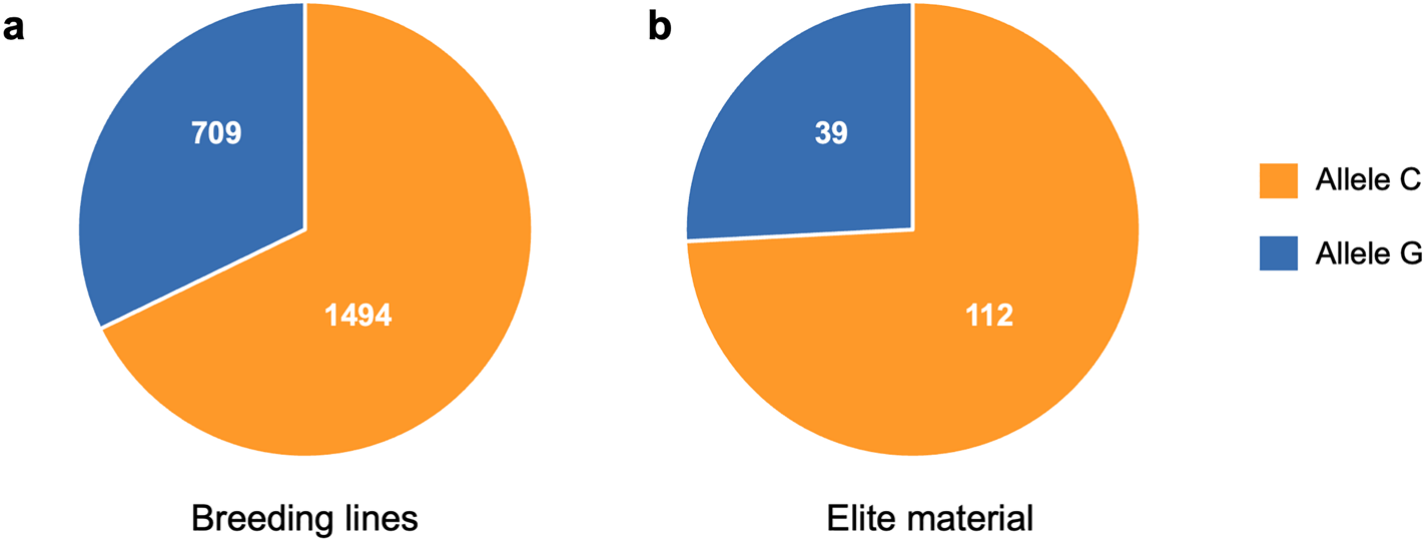
Pie charts showing the frequency distribution of two alleles of *S1A_581955638*, the SNP representing *qFLANG.1A.* (**a**) The frequency distribution in breeding lines from the South Dakota State University winter wheat breeding program, and (**b**) in a set of released cultivars and elite material from various HWW breeding programs of the US Great Plains region

The frequency of allele ‘C' for narrow and ‘G’ for wide flag leaf angle for marker *S1A_581955638* in the WWAM panel was 68.6% (177) and 31.4% (81), respectively. To study the spatial distribution of the two alleles in the HWW region, we used a large set of 2354 HWW lines consisting of 2203 breeding lines from SDSU preliminary or advanced trials and 151 Elite materials consisting of released HWW cultivars or lines tested in regional nurseries from different breeding programs. Among the 2203 breeding lines, 1,494 (68%) carried the ‘C’ allele, while 709 (32%) carried the ‘G’ allele (Fig. 5a). However, a slightly higher frequency of the ‘C’ allele was observed in Elite materials (74%) compared to breeding lines, whereas only 26% of lines carrying the ‘G’ allele (Fig. 5b).

### Development of a KASP assay to validate *qFLANG.1A* in spring wheat population

**Fig. 6:**
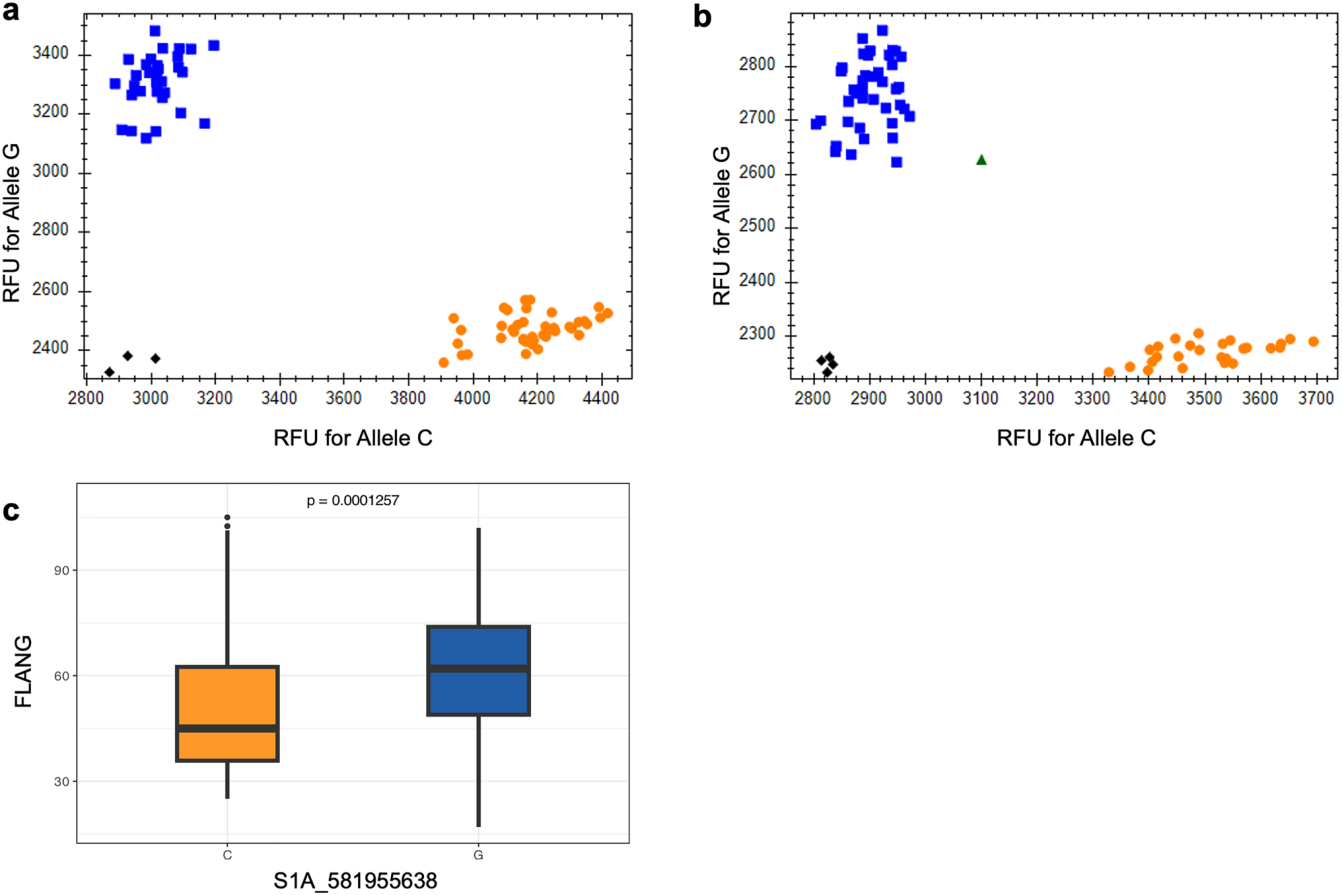
Allele discrimination plot for SNP *S1A_581955638 *using KASP assay evaluated on (**a**) a set of genotypes from the WWAM panel and **b** on a set of diverse spring wheat genotypes used for independent validation. **c** Boxplot comparing the FLANG among allelic groups of *qFLANG.1A* in an independent natural population

Considering the large effect of *qFLANG.*1A on flag leaf angle, a KASP assay was developed to facilitate marker-assisted selection of *qFLANG.*1A in the breeding programs*.* The KASP assay was tested on a set of 158 accessions from the WWAM panel, and the marker correctly discriminated the two alleles at SNP *S1A_581955638* (Fig. 6a). Further, the KASP assay was used to screen an independent set of 200 diverse spring wheat accessions described in materials (Fig. 6b; Supplementary Table S4). Subsequently, the FLANG data for these 200 accessions were compared for different allelic groups (Supplementary Table S4). The mean FLANG values were significantly different (*P* = 0.00013) with an average angle of 50.03 for the ‘C’ group and 61.47 for the ‘G’ group (Fig. 6c), confirming the significance of *qFLANG.*1A and the effectiveness of the KASP assay in spring wheat germplasm.

## Discussion

Flag leaf and its posture determine plant photosynthetic capacity and in turn wheat productivity (Zanella et al. 2023). Altering the flag leaf architecture to maximize photosynthate assimilation at the grain-filling stage is a desirable goal for wheat breeding programs. Various photosynthetic component traits including FLM showed genetic variation and were consistently heritable despite their quantitative nature. This makes them suitable for phenotypic selection, as required for breeding superior wheat cultivars (Poland 2015). We observed a wide range of variations in FLM traits (FLL, FLW, FLANG, FLR, FLA), plant height, days to heading (Table 1), and high heritability, consistent with previous reports (Chen et al. 2021; Wang et al. 2022). The high heritability for FLANG (0.94) in the current study was similar to or higher as compared to previous studies, indicating a significant potential for wheat canopy improvement (Chen et al. 2020, 2021).

Similar to previous studies, we also observed significant positive correlations (Fig. 1g) between plant height and FLL (0.40), FLA (0.16), and FLR (0.40), respectively (Hu et al. 2020; Gao et al. 2021; Sun et al. 2017; Liu et al. 2017; Chen et al. 2021; Li et al. 2021; Muhammad et al. 2021a, b). In addition to other factors, the observed correlations between height and FLL might be attributed to the effect of various genes involved in the brassinosteroid (BR) signaling pathway. Previous studies have shown BR pathway genes have a direct impact on leaf architecture and exhibit pleiotropic effects on plant height (Makarevitch et al. 2012; Hirano et al. 2017; Mantilla-Perez and Salas Fernandez 2017). BR-deficient (BRASSINOSTEROID INSENSITIVE 1 gene) or mutant phenotypes tend to exhibit small leaf angles with reduced height (Morinaka et al. 2006; Sakamoto et al. 2006). Mutants of the BRASSINOSTEROID-SIGNALING KINASE1 (BSK1) gene in rice also resulted in dwarf plants with erect leaves (Bai et al. 2007; Zhang et al. 2012). Sorghum plants with a mutated DWARF1 (DW1) are similar to BR-deficient rice mutants, with phenotypes that include a reduced plant height and leaf angle (Hirano et al. 2017). However, surprisingly we did not observe a significant correlation between FLANG and plant height, suggesting alternate pathways (phytohormones, auxin, ethylene, strigolactone, and gibberellin) may also affect the FLANG in wheat (Shimada et al. 2006; Zhao et al. 2013; Li et al. 2014, 2020a, b). Wang et al. (2020) have also suggested other signals, including circadian rhythm, biotic stresses, MAPK signaling, phosphatidylinositol signaling, and calcium-mediated signaling, may play important roles in regulating leaf angle plasticity; however, it needs further investigation.

The GWAS identified 36 MTAs (Supplementary Table S6) for FLM traits; however, we focused on nine stable MTAs that were consistently identified in multiple environments, making them more reliable and valuable for wheat breeding (Table 2, Supplementary Table S7). Out of six stable MTAs for FLL identified in the present study, three MTAs (*S2B_44931079, S3A_688732686,* and *S5A_570625938*) on chromosome 2B, 3A, and 5A are located in the vicinity of previously reported QTLs for FLL in recent studies (Wu et al. 2016; Liu et al. 2018a; Li et al. 2019; Yan et al. 2020c; Du et al. 2022). The other three genomic regions (MTAs, *S1B_653531690, S4A_665013137*, and *S4B_459361891*) associated with FLL are novel with no QTL previously reported in these regions. The identification of known and new genomic regions affecting FLL shows the robustness and consistency of our findings and validates the importance of these regions in controlling FLL.

In this study, we identified three stable genomic regions/MTAs for FLANG, namely *S1A_581955638*, *S5A_473723888*, and *S5B_247555050*, on chromosomes 1A, 5A, and 5B, respectively. Notably, all three genomic regions seem to be novel as no genes/QTLs affecting FLANG have been documented in their vicinity in wheat. The understanding of the genetic architecture of leaf inclination is limited in wheat ( Sun et al. 2017; Liu et al. 2018a; Chen et al. 2021). To date, only one gene, *TaSPL8*, has been cloned for FLANG in wheat using map-based cloning. It encodes a *SQUAMOSA PROMOTER BINDING-LIKE* (*SPL*) protein, which regulates lamina joint development through auxin signaling and the brassinosteroid biosynthesis pathway, resulting in the exhibition of erect leaves. Conversely, the genetics of flag leaf angle have been extensively studied in various cereal crop species (Mantilla-Perez and Salas Fernandez 2017). In maize, numerous genes associated with leaf angle including *LIGULELESS* (Bauer et al. 2004; Buescher et al. 2014), *ZmCLA4* (Zhang et al. 2014), and *BRASSINOSTEROIDDEFICIENT DWARF1* (Makarevitch et al. 2012) have been cloned. Similarly in rice, genes like *OsDWARF* (Hong et al. 2002), OsBRI1 (Li and Chory 1997; Yamamuro et al. 2000), *LAZY1* (*LA1*) (Li et al. 2007), *LEAF AND TILLER ANGLE INCREASED CONTROLLER* (*OsLIC*) (Sinclair and Sheehy 1999; Tanaka et al. 2009; Zhang et al. 2012), and *BRASSINOSTEROID UPREGULATED 1-LIKE1* (*OsBUL1*) (Jang et al. 2017), and in sorghum, *DWARF3* (*DW3*) (Knöller et al. 2010; Truong et al. 2015) and *DWARF1* (*DW1*) (Yamaguchi et al. 2016; Hirano et al. 2017) have been associated with the leaf inclination.

Among three MTAs, we identified a major and novel region (*qFLANG.1A*) with a consistently strong signal on chromosome 1A that explained 9–13% of the variation across different environments. Comparative synteny with rice and maize revealed no orthologs associated with canopy architecture in the region (581–587 Mb) harboring *qFLANG.1A* (Mantilla-Perez and Salas Fernandez 2017). To further validate the significance of this major region and facilitate MAS, we developed a KASP assay specifically for *qFLANG.1A* to discriminate the two alleles (Fig. 6a). Moreover, we were interested to validate the effect of *qFLANG.1A* beyond hard winter wheat to other classes of wheat. Hence, we used the KASP assay to discriminate the alleles for *qFLANG.1A* in a diverse set of spring wheat accessions that was randomly selected from a global core set of spring wheat. The spring wheat set was phenotyped for FLANG, and the KASP assay was robust for identifying the narrow and broad FLANG genotypes in an independent set, and the two allelic groups differed significantly (*P* = 0.00013) for FLANG. The independent validation in a diverse set provides strong evidence supporting the importance of this genomic region in regulating FLANG not only in winter wheat but also in spring wheat germplasm. Therefore, it becomes compelling to delve deeper into this region and identify the putative gene underlying this important and novel QTL.

Building upon this validation, the spatial distribution of *qFLANG.1A* alleles across a substantial dataset comprising 2354 HWW lines, including SDSU breeding lines and elite material from various breeding programs evaluated in regional nurseries across the Great Plains, revealed a notable pattern. We observed a higher frequency of alleles associated with narrow leaf angle in all breeding lines similar to the allele distribution observed in our WWAM panel. Remarkably, this consistent trend was also evident in the elite material/varieties, indicating a widespread distribution of these alleles across the Great Plains. This suggests that narrow leaf angle traits have been indirectly selected during the breeding process, likely as part of efforts to enhance yield potential. However, it is noteworthy that despite the strong association with narrow leaf angle traits, we found only one report of QTL (*QYld.crc.1A*) affecting yield and yield-related traits mapped close to *qFLANG.1A* (Cuthbert et al. 2008).

We identified seven high-confidence genes of interest for *qFLANG.1A*. The most interesting among these seven genes is *TraesCS1A03G1030200* encoding glucan endo-1,3-beta-glucosidase 3. The most significant and stable SNP for *qFLANG.1A* (*S1A_581955638*) was mapped within the gene *TraesCS1A03G1030200*. Although we did not find the role of this predicted protein in the regulation of flag leaf angle, a recent study on mapping flag leaf angle has delimited an important QTL for FLANG on chromosome 4B to a 5 Mb region and identified seven candidate genes based on differential expression and one of the seven genes encodes a beta-glucosidase protein (Zhang et al. 2024). This suggests a putative role of this gene family in the regulation of FLANG and supports further investigation. Another gene of interest is the F-box family protein coding gene (*TraesCS1A03G1026000*) identified in this region, which has been reported to be responsible for plant growth and development by regulating leaf growth and cell division, influencing cell numbers and consequently impacting leaf size (Yan et al. 2020a, b, c; Kong et al. 2023). Another gene identified encodes typical receptor-like protein kinases (RLKs) proteins that regulate plant growth, development, and stress responses (Cui et al. 2022). However, some reports have shown that RLKs are involved in the *BRASSINOSTEROID INSENSITIVE 1 (BRI1) gene* pathway, which mediates the BR signal to regulate cell elongation required for normal growth and development of the plant (Hothorn et al. 2011; She et al. 2011; Cui et al. 2022). We did not identify any annotated gene in the candidate region that was directly related to the BR pathway, which regulates leaf angle in many crops (Mantilla-Perez and Salas Fernandez 2017). The lack of BR pathway genes in the candidate region suggests the role of alternate pathways in regulating leaf angle. Furthermore, the nonsignificant correlation between FLANG and plant height in our study also suggests the possibility of alternate mechanisms affecting FLANG. Various phytohormones, including auxin, ethylene, and gibberellin, may play a role in the regulation of FLANG and need to be further investigated.

By studying the genomic region spanning *qFLANG.5A*, we identified several genes that may potentially affect FLANG. The gene *TraesCS5A03G0658100* encodes a squamosa promoter-binding-like protein, which is similar to SPL protein exhibiting an erect leaf phenotype and regulates lamina joint development by affecting the auxin response and BR biogenesis pathway in wheat (Liu et al. 2019). Additionally, the SQUAMOSA PROMOTER BINDING-LIKE (SPL) protein is related to an erect leaf phenotype in maize and rice (Moreno et al. 1997; Lee et al. 2007). Another gene, *TraesCS5A03G0644800,* encodes a SHAGGY-like kinase protein, which is known to be involved in hormonal signaling networks and required for growth and stress responses. This protein is a key player in the brassinosteroid (BR) signaling pathway and affects various developmental processes and responses to environmental factors (Bittner et al. 2015; Kloc et al. 2020). Additionally, gene *TraesCS5A03G0657600* encodes the ETHYLENE INSENSITIVE 3-like protein which plays a crucial role in regulating a range of functions including leaf development, senescence, and germination stimulation (Dubois et al. 2018). Further studies to validate these candidate genes will help unravel their precise roles and mechanisms regulating FLANG.

In conclusion, this is the first major study dissecting the genetic architecture of FLM traits in US winter wheat where multi-environment GWAS identified 36 MTAs for FLM traits. Nine genomic regions were stable across environments, and three each for FLL and FLANG are novel and have not been previously reported. Further investigation and independent validation of *qFLANG.1A*, a major QTL associated with FLANG using KASP markers, suggested its importance in both winter and spring wheat panels and its potential use in marker-assisted selection (MAS) in the wheat breeding programs. Fine mapping of this region will be useful to gain a more detailed understanding of the genetic regulations of FLANG in wheat and its potential implications in wheat breeding programs. Further, the SNP markers linked to the stable regions identified in this study could also be incorporated into multivariate genomic selection models for the enhancement of the photosynthetic efficiency of wheat. This research opens avenues for both practical breeding applications and further exploration of genomic regions influencing FLM traits in winter wheat.

## Supplementary Information

Below is the link to the electronic supplementary material.

Supplementary file1 (PDF 200 KB)

Supplementary file2 (PDF 117 KB)

Supplementary file3 (XLSX 36 KB)

## Data Availability

The datasets generated in the study are either presented in the supplementary information associated with the article or can be provided on request to the corresponding author.
