# Supplementary material for: Characterization of flag leaf morphology identifies a major genomic region controlling flag leaf angle in the US winter wheat (Triticum aestivum L.)
Source: Theor Appl Genet. 2024 Aug 14;137(9):205. doi: 10.1007/s00122-024-04701-1 (PMC11324803; doi:10.1007/s00122-024-04701-1)

Characterization of flag leaf morphology identifies a major genomic region controlling flag leaf angle in the US winter wheat (*Triticum aestivum* L.)

Supplementary Figure S1. (a). Scree plot for the principal component analysis using 14,537 single nucleotide polymorphisms (SNPs) and (b) Evanno plot of Delta-K statistic from the STRUCTURE analysis

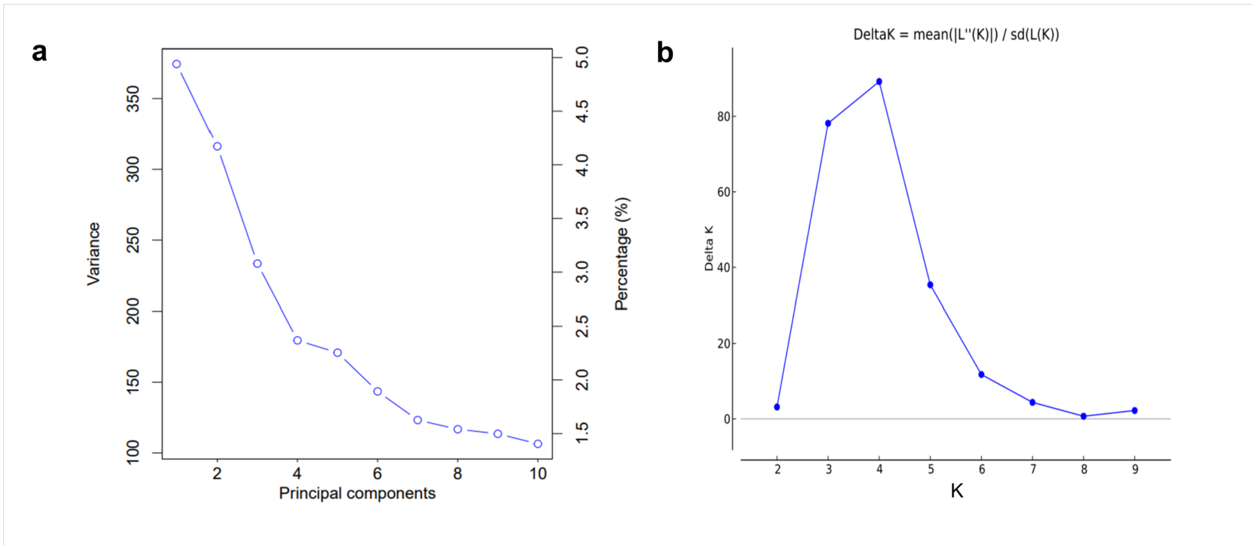

Supplement: Supplementary file 1 — Supplementary file1 (PDF 200 KB) [file 122_2024_4701_MOESM1_ESM.pdf]
