# Supplementary material for: Characterization of flag leaf morphology identifies a major genomic region controlling flag leaf angle in the US winter wheat (Triticum aestivum L.)
Source: Theor Appl Genet. 2024 Aug 14;137(9):205. doi: 10.1007/s00122-024-04701-1 (PMC11324803; doi:10.1007/s00122-024-04701-1)

Supplementary Figure S2. The neighbor joining tree for 270 lines based on the genotypic data.

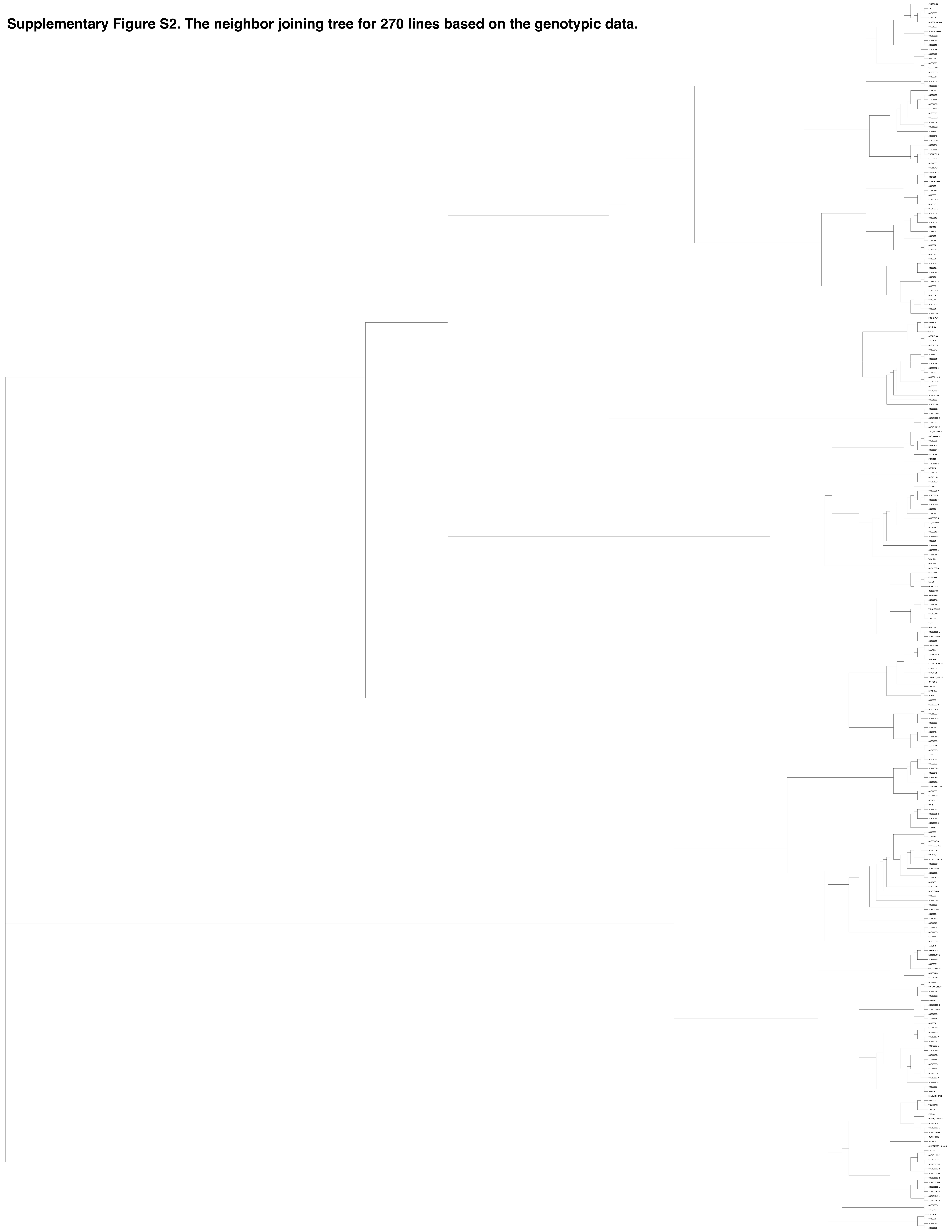

Supplement: Supplementary file 2 — Supplementary file2 (PDF 117 KB) [file 122_2024_4701_MOESM2_ESM.pdf]
